# Seroprevalence of *Toxoplasma gondii* in White-Tailed Deer (*Odocoileus virginianus*) in New York State

**DOI:** 10.3390/pathogens14010030

**Published:** 2025-01-03

**Authors:** Emily D. Ledgerwood, Jason D. Luscier

**Affiliations:** Department of Biological and Environmental Sciences, Le Moyne College, Syracuse, NY 13214, USA; lusciejd@lemoyne.edu

**Keywords:** toxoplasmosis, wildlife disease, one health, seropositive, parasite, urbanization, modified agglutination test, TgMAT, domestic cat, *Felis catus*

## Abstract

The parasitic protozoa, *Toxoplasma gondii* (*T. gondii*), is a model organism for one health because of its wide-ranging impacts on humans, wildlife, and domestic animals. Intermediate hosts, including white-tailed deer (*Odocoileus virginianus*), have been implicated in its maintenance. Prior analysis of *Toxoplasma gondii* seroprevalence in New York State deer focused on rural areas; however, the high density of domestic cats (*Felis catus*) in urban areas has been implicated in its spread amongst deer. To address this, the seroprevalence of *Toxoplasma gondii* was assessed across two suburban and urban areas with known deer overabundance in Onondaga and Suffolk County. Here, domestic cats are the only likely definitive host. Between 2019 and 2023, serum from culled deer was collected, and *Toxoplasma gondii* seropositivity was determined using the modified agglutination test. Overall seroprevalence was 49.31% (*n* = 144) but was significantly higher in Onondaga (64%) compared to Suffolk County (36%), despite similarities between these two regions. Deer from Onondaga also had higher antibody titers. These data suggest that although urbanization may be a predictor of *Toxoplasma gondii* seropositivity in deer, there are additional contributing factors. Overall, this study emphasizes the need for continued surveillance in intermediate hosts and informs public health and wildlife management decisions aimed at limiting the impact of *Toxoplasma gondii*.

## 1. Introduction

*Toxoplasma gondii (T. gondii)* is a ubiquitous parasitic protozoan with worldwide distribution. Reproduction of the parasite is restricted to members of the Felidae family, making felids and domestic cats (*Felis catus*) the definitive hosts responsible for producing and spreading highly stable oocysts capable of surviving in the environment for years [1,2]. The global seroprevalence of *T. gondii* is generally higher in native felids than domestic cats, with the exception of the United States, where the estimated seroprevalence of domestic cats and native felids (e.g., bobcats [*Lynx rufus*]) is more comparable [3]. Urban areas tend to have particularly high densities of domestic cats, in many cases representing > 100 times the densities of native felids in more natural habitats [4]. These highly inflated population densities of domestic cats increase the *T. gondii* seroprevalence in some wildlife species in urban areas [5,6,7,8]. Therefore, the spread of *T. gondii* in cities is of noteworthy concern for both urban wildlife and human health. Although toxoplasmosis, the disease caused by *T. gondii*, is generally asymptomatic in humans, *T. gondii* is considered an important cause of foodborne illness, and infection in immunocompromised individuals or during pregnancy can result in life-threatening or severe congenital complications, respectively [9,10].

Given the potential for infection in humans, coupled with the growing implications for infection in wildlife, particularly marine mammals, monitoring the seroprevalence in sentinel species is of increasing importance [11,12]. White-tailed deer (*Odocoileus virginianus*, WTD) have been implicated in the maintenance, spread, and amplification of a variety of zoonotic pathogens, including *T. gondii* [13,14,15,16]. In addition to being the most common wild cervids in the United States, WTD are well suited for study due to their abundance in both rural and urban settings [12,17]. They are able to survive in a variety of habitats but thrive in those that provide both plant and woody vegetation at the interface between open agricultural fields and forests, consistent with the edge landscaping often found in suburban developments. Lack of predator pressure and ample food supply has resulted in an overabundance of deer in many regions across the United States. In New York State, areas of substantial development are paired with restrictions on hunting, giving rise to hot spots of deer overabundance in several counties, most notably Onondaga County in Central New York and Suffolk County located downstate [18].

In these counties, a suburban-to-urban gradient exists that provides opportunities for WTD, wildlife, domestic animals, and humans to interact. Given the role of the domestic cat in oocyst shedding, the impact of urbanization on seroprevalence in WTD and other species has been explored, with conflicting results [11,12,19]. In New York, only a handful of studies have assessed *T. gondii* seroprevalence in WTD despite a consistently high deer population [11,17]. To address these gaps, our objective was to determine the seroprevalence of *T. gondii* in WTD across two counties in New York State, each with a documented overabundance of WTD in addition to areas classified as suburban and urban [20]. We hypothesized that serostatus would be consistent across counties, but that seroprevalence would be elevated in urban areas where cat density is suggested to be higher [21].

## 2. Materials and Methods

### 2.1. Study Area

**Figure 1:**
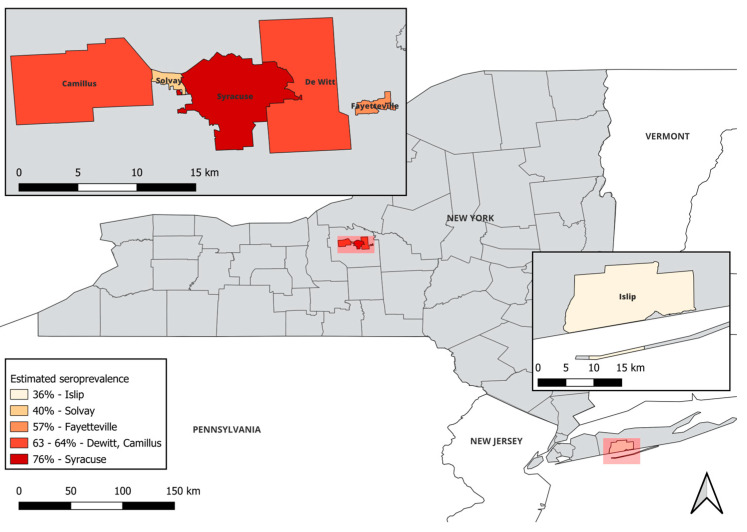
*Toxoplasma gondii* seropositivity in white-tailed deer does not correlate with population density. Deer samples were obtained across five locations in Onondaga County (upper left inset) and one location in Suffolk County (lower right inset) and *T. gondii* seroprevalence was determined. This map was created using QGIS version 3.32.3-Lima [30]. Human population densities of residents (res./km^2^) were computed based on the numbers of people within a 10 km radius of the geographic center of each municipality [29].

Within the state of New York, there are several counties with municipalities currently facing white-tailed deer (WTD) overabundance [20]. These counties are widely distributed across the state and include counties in Downstate (Suffolk, Westchester, and Orange), Central (Onondaga), Western (Genesee and Erie), and the Capital Region (Saratoga and Albany) of New York. In this study, we sampled across two of these counties, Onondaga and Suffolk (Figure 1). Onondaga County, in Central New York, is an isolated area of overabundance compared to Suffolk County, which exists in the largest region of deer density in the state. Both counties have implemented deer culling programs over the past 10 years. During both 2022 and 2023, 0.7–1.2 bucks/km^2^ and 0.7–1.5 antlerless deer/km^2^ were taken in Onondaga County, and 0.7–1.2 bucks/km^2^ and 0.7–1.2 antlerless deer/km^2^ were taken in Suffolk County [22].

Onondaga County is situated in the Ontario lowlands ecoregion due to its proximity to Lake Ontario. This region is characterized by consistent lake-effect cloud cover and precipitation and historically was dominated by beech (*Fagus grandifolia Ehrh*.) and sugar maple (*Acer saccharum Marshall*) [23]. Overall, the county is highly fragmented into agricultural regions, urban centers (e.g., the Syracuse metropolitan area), and small remnant forest patches, making it an ideal landscape for WTD population growth. Suffolk County is situated on the eastern part of Long Island and is characterized by a climate moderated by the surrounding ocean and by stunted arborescent growth of pines (*Pinus* spp. L.) and oak (*Quercus* spp. L.). Multiple small urban areas are scattered about the county, including the Hamptons in the East and Islip, Melville, etc. in the West. The fragmented landscapes in each of these counties are ideal for WTD population growth.

The only native felid in New York is the bobcat (*Lynx rufus*). Bobcats do not occur in Suffolk County and are thought to rarely occur in Onondaga County. Regardless, bobcats avoid high densities of human populations in New York [24]. Additionally, *T. gondii* prevalence in wild felids (e.g., bobcats) is lower in North America compared with other regions of the world [3]. The domestic cat is the most likely reservoir for *T. gondii* in our study area. Domestic cat population densities in cities have been estimated to be as high as >1500/km^2^, whereas bobcat population densities are generally <0.01/km^2^ [4].

### 2.2. Sample Collection

Blood samples were obtained from a total of 144 white-tailed deer (WTD) representing two populations in New York State: Onondaga County (*n* = 69) and Suffolk County (*n* = 75). The specific municipalities that enacted a cull management scheme as part of regional efforts to reduce WTD damage in urban and suburban environments included Syracuse, Camillus, Dewitt, Fayetteville, and Solvay in Onondaga County and Islip in Suffolk County. In these municipalities, deer were killed by sharpshooters and USDA-APHIS, Wildlife Services during the winter months of 2019, 2020, and 2023 (Onondaga County) and 2020 and 2023 (Suffolk County). Each municipality participating in this cull management of WTD obtained Deer Damage Permits from the New York State Department of Environmental Conservation. Sampling was restricted to deer harvested as part of these culling programs (i.e., we did not have any control over the direct harvest of deer). Samples were collected across multiple seasons, which may help to account for year-to-year variations in deer abundance, etc. No deer was killed for the purposes of this study.

### 2.3. Sample Processing

Blood samples were obtained via cardiac puncture within 6 h of death and collected into Vacutainer tubes (Becton Dickinson, Franklin Lakes, NJ, USA) before storing at 4 °C. Samples were processed by allowing them to coagulate at room temperature for 30 min, followed by centrifugation at 1600× *g* for 10 min. Most samples were processed within 72 h. Serum was transferred to a cryovial and stored minimally at −20 °C until serology testing.

### 2.4. Serology

To detect anti-Toxoplasma IgG antibodies in serum, the commercially available Toxoplasma Whole-cell Antigen for Modified Agglutination Test was performed according to the manufacturer’s instructions (TgMAT, University of Tennessee Research Foundation, Memphis, TN, USA) [25,26]. The kit-provided antigen consisted of formalin-fixed tachyzoites harvested from human foreskin fibroblast cultures infected with the *T. gondii* RH strain. Briefly, serum samples were diluted 1:25 in phosphate buffered saline (PBS) before combining 25 μL of the diluted sample with an equal volume of PBS in a 96-well v-bottom plate. Samples and controls were serially diluted to 1:200 and 1:3200, respectively. Then, a *Toxoplasma* antigen mixture was prepared by diluting formalin-fixed tachyzoites in an alkaline buffer (7.01 g/L NaCl, 3.09 g/L boric acid, 1 g/L sodium azide, 0.05% sodium hydroxide). The alkaline buffer was pH adjusted to 8.95 before adding 4 g/L bovine plasma albumin and supplemented with 2% Evans blue dye and 2-mercaptoethanol to create the *Toxoplasma* antigen mixture. Using a multi-channel pipette, 25 μL of the antigen mixture was added to each well and mixed. Plates were sealed and incubated at 37 °C for 16–20 h before assessing positivity. The presence of a pellet at the bottom of the well indicated a sample was negative for anti-Toxoplasma IgG serum antibodies. *T. gondii* seroprevalence was reported as a titer score. Samples with titers of 1:25 or greater were determined seropositive as defined by others [27].

### 2.5. Data Analysis

Variation in the proportion of seropositive animals by county was evaluated using multiple logistic regression analysis based on a likelihood ratio test (PRISM 6). Confidence intervals were calculated using the method of Clopper and Pearson (PRISM 6) [28]. Direct detection rates in seronegatives and seropositives were compared using Fisher’s exact test (PRISM 6). To assess associations between seroprevalence and year, age, or sex, Fisher’s exact test was used (PRISM 6). To evaluate for a simple linear relationship between human population densities and seroprevalence among the specific municipalities, a Pearson correlation coefficient was computed (R 3.6.2). A value of *p* ≤ 0.05 was considered statistically significant for all tests.

## 3. Results

### 3.1. Seroprevalence of Toxoplasma gondii in White-Tailed Deer in New York State

The seroprevalence of *T. gondii* in WTD across two counties in New York State was determined. A total of 69 WTD were culled in Onondaga County across 2019, 2020, and 2023, with 63.77% having *T. gondii*-specific antibodies (Table 1). Seroprevalence ranged from 61.53–72.22% across the three years, suggesting consistency across hunting seasons. In contrast, only 36% of deer tested in Suffolk County were positive for *T. gondii*-specific antibodies in 2020 (22.22%) and 2023 (37.88%) (Table 2). It is important to note that the 2020 Suffolk sample size was small, with only nine samples collected. In all years sampled, the seroprevalence rate was significantly higher in Onondaga compared to Suffolk County (*p* = 0.0014).

In addition to a greater percentage of WTD being seropositive in Onondaga County compared to Suffolk County, deer harvested from Onondaga also exhibited higher antibody titers (Table 3). Over 43% of WTD from Onondaga County had high antibody titers (1:200) compared to only 7.4% of WTD from Suffolk County (*p* = 0.0012, Table 3). In contrast, only 25% of WTD from Onondaga County had antibody titers at baseline seropositivity (1:25) compared to 59.3% of Suffolk County samples (*p* = 0.0056, Table 3). Together, these data suggest that differences in seroprevalence exist across New York State and that the seroprevalence and titer of *T. gondii*-specific antibody are higher in WTD in Onondaga County compared to Suffolk County.

### 3.2. Juvenile Deer Are More Likely to Be Seropositive in Onondaga County

The rate of seropositivity was consistently higher in adult deer compared to juvenile deer across both counties; however, it was only statistically significant in Suffolk County in 2023 (Table 1 and Table 2). Comparisons between counties revealed several differences. Firstly, although females, males, adults, and juveniles from Onondaga County had a higher rate of seropositivity compared to Suffolk County, the difference between rates observed in each county was particularly striking in juvenile deer. During the study period, the seroprevalence of *T. gondii* in juveniles from Onondaga County was 55% compared to only 6.25% of juveniles from Suffolk County (Table 1 and Table 2, *p* = 0.0035). Onondaga juveniles also had higher antibody titers. Of those deer that were seropositive, 54.5% of juvenile deer from Onondaga County had high MAT titers compared with 0% of juveniles from Suffolk County (Table 3). Together, these data suggest that juveniles are more likely to be infected in Onondaga County compared with Suffolk County.

### 3.3. Human Population Density Does Not Consistently Correlate with Percent Seropositivity

In this study, samples were collected from five towns across Onondaga County, including both urban and suburban sites. Of these sites, the city of Syracuse was the most densely populated (3237 residents/km^2^) and was found to have the highest percent positivity (76.47%) (Figure 1 and Table 4) [29]. Two of the five sites, Camillus and Dewitt, had similar but lower population densities (374 and 389 residents/km^2^, respectively), with comparable lower percent seropositivity (64.29% and 63.16%, respectively). However, a third suburban site, the town of Fayetteville, had a percent seropositivity rate of 57.14%, despite being more densely populated with 959 residents/km^2^. Furthermore, the town of Solvay had the lowest percent positivity in the county (40%) paired with the second highest population density in Onondaga County (1355 residents/km^2^). It is important to note that the sample size for Solvay was significantly smaller than the other sites (*n* = 5); a potential limitation in the data. The collection sites for Suffolk County were limited to two state parks within the town of Islip. Islip had the lowest seropositivity rate (36%) across all sites tested in New York, despite a moderate population density (765 persons/km^2^). Although trends seemed apparent, the Pearson correlation coefficient was 0.41 (*p*-value = 0.42), indicating little evidence given our data for a correlation between human population densities and seroprevalence. These data suggest that although human population density could be a predictor of *T. gondii* seropositivity in WTD, there are additional contributing factors.

## 4. Discussion

In this study, the overall seroprevalence of *T. gondii* in sampled white-tailed deer (WTD) from New York State was estimated to be 49.31% (71/144). This is in line with other reports of *T. gondii* seroprevalence in WTD across the United States (22.5–74.4%) and in New York specifically (38.5%) [11,12,27,31]. Members of the Cervidae family are estimated to have a higher seroprevalence of *T. gondii* in the United States (40.42%) and North America (32.21%) in general, compared to other regions of the globe, notably Asia (12.72%), with the overall global seroprevalence of *T. gondii* in WTD estimated to be 39.4% [32]. Here, seropositivity was determined in WTD from two counties, Onondaga County and Suffolk County. Many similarities exist within areas across these counties, including human population levels and deer overabundance. Despite this, we observed striking differences between them, with Onondaga having an overall seroprevalence of 63.77% compared to Suffolk at 36%. One potential explanation for this difference could reflect how the deer were harvested. In Onondaga County, the deer were from open, county-wide harvests, compared to controlled park hunts in Suffolk County. The potential for population selection to influence seropositivity was previously introduced following observed differences in WTD seropositivity from a controlled park hunt in Minnesota (30%) compared to an open, state-wide harvest conducted in Pennsylvania (60%) [31,33]. Together, these findings suggest implications for WTD management plans. If an objective is to reduce the persistence or spread of disease, it may be more beneficial to conduct county-wide harvests. Suffolk County, and Islip specifically, is surrounded by areas of WTD overabundance compared to Onondaga County, which is relatively isolated. This indicates that while deer are a good species for monitoring *T. gondii* presence, deer density is unlikely to contribute to the overall level of environmental contamination.

In addition to differences in overall seropositivity between counties, WTD from Onondaga County had significantly higher antibody titers when compared with WTD from Suffolk County. Others have shown that a MAT titer of 1:200 correlated with a rate of viable parasite isolation in 10.7% of samples, whereas MAT titers of ≥1:3200 resulted in 50% detection, indicating both the specificity and sensitivity of the MAT in WTD [17]. Following infection, immunoglobulin M (IgM) antibodies are generated before seroconverting to immunoglobulin G (IgG). This process takes roughly 10–14 days. In cats, *T. gondii*-specific IgG levels remain high for ~2 months before falling; however, the cat will remain positive for years [34]. In the MAT test, the addition of β-mercaptoethanol (2-ME) destroys IgM antibodies to facilitate detection of *T. gondii*-specific IgG. The difference in titer across counties may potentially reflect repeated exposures in Onondaga County, reflective of increased environmental oocyst contamination in the area. Since the MAT does not distinguish between acute and chronic infections, we cannot rule out the possibility that there was a higher rate of acute infections in WTD from Onondaga at the time of the study, although this seems unlikely. A targeted study examining soil contamination across these regions might be of value, although others cite challenges with the sensitivity and reproducibility of this approach [35].

In this study, a relationship between human population density and *T. gondii* seropositivity in WTD was not consistently observed. This is in contrast to other studies, including a prior study conducted in Northeastern Ohio that found urban deer were 2.98 times more likely to be seropositive compared to suburban deer, with household density serving as a significant predictor of serostatus [7,12]. This question has been addressed in other species of wildlife with similar results [7,36,37,38,39]. It has been suggested that a high human population density may be associated with an increase in the domestic cat population. Given that felids are the only known definitive host for *T. gondii*, with the domestic cat likely serving the most consequential role, urban wildlife may be at increased risk for exposure [40]. Still, other factors, including the contribution of wild felid species (albeit likely minimal or absent in these counties), the potential for outdoor domestic cats in rural areas to have increased exposure to *T. gondii*, and the influence of WTD range, particularly those inhabiting at the interface between suburban and urban areas, cannot be discounted [11]. Although a direct correlation between population density and seropositivity cannot be drawn in this study, the data do suggest that population density is an important factor. Other reports that population density does not correlate with seropositivity rate in WTD exist, including two studies performed in the US, one of which was also in New York State [11,19]. However, in that study, the majority of samples came from rural towns in New York State with a median human population density of 25 residents/km^2^ [11]. They reported a seroprevalence of 38.5% compared to our 49.31% using the same MAT test to determine seropositivity [11]. In this study, WTD were harvested from five towns in Onondaga County with differing population densities to address the limitations of the prior study conducted in New York. When compared to our study, which assesses seroprevalence primarily in suburban and urban areas, a role for human density is supported. The city of Syracuse had both the highest population density (3237 residents/km^2)^ and the highest percent positivity (76.47%). However, the town of Solvay had the lowest seropositivity in the county despite a significant population (1355 residents/km^2^). Only five deer were harvested from this town, all during the 2023 season. Together, these studies emphasize the importance of collecting a sufficient number of samples, across a rural to urban gradient, for more than one season.

The high seropositivity in WTD observed in this study raises significant public health concerns given that *T. gondii* consistently ranks in the top five pathogens associated with hospitalization and death attributed to foodborne illness in the United States [41]. In addition to being a popular game meat in the United States and elsewhere, many states have venison donation programs to feed the hungry [42]. The Quality Deer Management Association reported that their members alone shared or donated 13.2 million meals from the roughly 6 million deer harvested in 2019 [43]. In Onondaga County, there is a significant outreach program for venison donations. The New York State Department of Environmental Conservation partners with local organizations to make nearly 40 tons of venison available to needy families on an annual basis. One specific deer management effort took place in Onondaga County between December 2021 and March 2022 across Syracuse, Camillus, Solvay, DeWitt, Fayetteville, and Manlius. In the city of Syracuse alone, 92 deer were culled, resulting in the donation of 2373 pounds of venison, amounting to 9492 local meals [44]. Intermediate hosts are infected with *T. gondii* in two primary ways: ingestion of tissue cysts in raw or undercooked meat of infected animals or ingestion of cat-excreted environmental oocysts on vegetables or in contaminated water [45]. Following ingestion of oocysts, the resulting tissue cysts then localize primarily to muscular and neural tissues, persisting in those tissues for the life of the host [46]. To avoid ingestion of tissue cysts, venison should be cooked to a minimum internal temperature of 71 °C before consumption [47]. Additional methods of preparation include salting, curing (for jerky), and smoking; however, these methods have not proven reliable at inactivating tissue cysts [47,48]. Tissue cysts are killed with freezing, but the temperature and timing must be carefully controlled, and there is a range of recommendations from −12 °C for 2 days to −20 °C for 3 days [49]. Although infected WTD likely contribute primarily to disease in humans via consumption of undercooked venison, there are reports of toxoplasmosis following the slaughtering and handling of visceral tissues [50,51,52,53,54,55,56]. In one study, handling and cleaning of infected carcasses by hunters was linked to ocular toxoplasmosis [50]. The DEC and the Cornell University Center for Conservation Social Science conducted a survey that was distributed to 25,750 property owners in New York between 2018 and 2020. Accounting for a 42% response rate, 31% of respondents identified as hunters, yet only 3% of New York residents are licensed hunters [57]. This highlights existing challenges with educational outreach.

Toxoplasmosis has been designated as a one health disease with wide-ranging impacts on both terrestrial and oceanic ecosystems and clear health implications for humans, wildlife, and domestic animals [58,59,60]. *T. gondii* has resulted in seven outbreaks of human toxoplasmosis in the U.S. between 1968 and 2018, four of which were attributed to the consumption of venison [61]. Beyond this, *T. gondii* infection in farmed animals may lead to a significant economic burden, particularly in sheep and goats, where it serves as a major cause of reproductive losses [62]. Intergrazing between WTD and agricultural herds does occur, and monitoring the seroprevalence in WTD may be indicative of infection levels in locally farmed animals. Measures such as housing agricultural animals indoors or restricting access by cats could help to limit transmission [62]. Potential interventions to reduce the overall disease burden of *T. gondii* have been reviewed by others [63]. Here we outline strategies to specifically limit the impact of infected WTD (Table 5).

In addition to acute disease, *T. gondii* is associated with behavioral changes in infected mammals, including slower reaction times and increased risk-taking [64,65,66]. Wild gray wolves (*Canis lupus*) that reside in regions with high cougar (*Puma concolor*) density were more likely to make high-risk decisions [67]. A study spanning three decades of blood collection and field observations of the spotted hyena (*Crocuta Crocuta*) found that, compared to uninfected cubs, seropositive cubs had a shorter minimum approach distance to lions (*Panthera leo*), which was associated with a higher mortality rate in those animals [68]. Others have observed that the seroprevalence of *T. gondii* is higher in road-kill animals than in culled animals [69]. Both Onondaga and Suffolk counties consistently lead the state in animal crashes, with the city of Syracuse reporting an average of 40 motor vehicle collisions with deer annually from 2019–2021 [44]. A high regional seroprevalence may call for additional signage and monitoring efforts to limit vehicle collisions (Table 5). In this study, we assessed seropositivity across the following subgroups: juvenile, adult, male, and female. Our data were consistent with previous studies that have suggested that *T. gondii* seroprevalence increases with age in some species [11,12,27,70]. In Onondaga County, seropositivity was similar across different subgroups, ranging from 55–67% (Table 1). This was in contrast to what we observed in Suffolk County, where the juvenile subgroup substantially deviated from other subgroups (6.25% versus 34–44%). Our finding that juvenile deer from Suffolk were less likely to be seropositive than juvenile deer from Onondaga (6.25% and 55%, respectively) may indicate that *T. gondii* infection has influenced population-wide behavioral shifts. It is tempting to speculate that the high rate of seropositivity observed in Onondaga County is the result of an exposure feedback loop wherein WTD are less risk averse and more likely to come in close proximity to homes, potentially with their young, increasing the frequency of exposure to environmental oocysts. Together, these observations suggest that toxoplasmosis has ecological significance even in situations when intermediate hosts do not exhibit overt acute disease.

Overall, this study reveals that *T. gondii* seroprevalence in white-tailed deer is high in urban and suburban areas across New York State and increases with age. Native felid presence is unlikely in the counties sampled, highlighting the role of the domestic cat in the epidemiology of *T. gondii* in urbanized areas. Overall, this study emphasizes the need for continued surveillance in wild intermediate hosts coupled with public outreach and educational campaigns to limit the spread and impact of *T. gondii* on both humans and wildlife.

## Figures and Tables

**Table 1:** Seroprevalence of *Toxoplasma gondii* in white-tailed deer in Onondaga County, New York by year, sex, and age.

Years	Category	No. Positive/No. Tested	% Seroprevalence (95% CI)	*p*-Value
2019–2023	Total	44/69	63.77 (51.31–75.00)	0.76
	Age			
	Juvenile	11/20	55.00 (31.53–76.94)	0.43
	Adult	33/49	67.35 (52.46–80.05)	0.76
	Sex			
	Female	23/40	57.50 (40.89–72.96)	0.72
	Male	19/28	67.86 (47.65–84.12)	0.78
2019	Total	13/18	72.22 (46.52–90.31)	
	Age			
	Juvenile	6/9	66.67 (29.93–92.52)	1
	Adult	7/9	77.78 (39.99–97.19)	
	Sex *			
	Female	4/6	66.67 (22.28–95.68)	1
	Male	8/11	72.73 (39.03–93.98)	
2020	Total	15/26	61.54 (38.67–78.88)	
	Age			
	Juvenile	2/6	33.33 (4.33–77.72)	0.35
	Adult	13/20	65.00 (40.78–84.61)	
	Sex			
	Female	8/16	50.00 (24.65–75.35)	0.43
	Male	7/10	70.00 (34.76–93.33)	
2023	Total	16/25	64.00 (42.52–82.03)	
	Age			
	Juvenile	3/5	60.00 (14.66–94.73)	1
	Adult	13/20	65.00 (40.78–84.61)	
	Sex			
	Female	12/18	61.11 (86.66–40.99)	1
	Male	4/7	57.14 (18.41–90.10)	

* Sex unknown in one sample. Note: Statistical analysis was performed using the Fisher’s exact test. The sex of one animal in 2019 was not recorded and was excluded in the analysis.

**Table 2:** Seroprevalence of *Toxoplasma gondii* in white-tailed deer in Suffolk County, New York by year, sex, and age.

Years	Category	No. Positive/ No. Tested	% Seroprevalence (95% CI)	*p*-Value
2020–2023	Total	27/75	36 (25.23–47.91)	0.47
	Age			
	Juvenile	1/16	6.25 (30.23–0.16)	1
	Adult	26/59	44.07 (31.16–57.60)	0.69
	Sex			
	Female	18/52	34.62 (21.97–49.09)	1
	Male	9/23	39.13 (19.71–61.46)	0.13
2020	Total	2/9	22.22 (2.82–60.01)	
	Age			
	Juvenile	0/3	0.00 (0.00–70.76)	0.5
	Adult	2/6	33.33 (4.327–77.72)	
	Sex			
	Female	2/5	40.00 (5.27–85.34)	0.44
	Male	0/4	0.00 (0.00–60.24)	
2023	Total	25/66	37.88 (26.22–50.67)	
	Age			
	Juvenile	1/13	7.69 (0.20–26.03)	0.0124 *
	Adult	24/53	45.28 (31.56–59.55)	
	Sex			
	Female Male	16/47 9/19	34.04 (20.86–49.31) 47.37 (24.45–71.14)	0.403

* Denotes significance, *p* ≤ 0.05. Note: Statistical analysis was performed using Fisher’s exact test.

**Table 3:** Anti-*Toxoplasma gondii* antibody titers as determined by modified agglutination test (baseline 1:25) in seropositive white-tailed deer across counties in New York.

Deer	No. Positive/	No. with MAT Titers			
	No. Tested	25	50	100	200
Onondaga					
Total	44/69	11 (25%)	9 (20.5%)	5 (11.4%)	19 (43.2%)
Adult	33/49	9 (27.3%)	8 (24.2%)	3 (9.1%)	13 (39.4%)
Juvenile	11/20	2 (18.2%)	1 (9.1%)	2 (18.2%)	6 (54.5%)
Male	19/28	5 (26.3%)	1 (5.3%)	4 (21.1%)	9 (47.4)
Female	24/40	6 (25%)	8 (33.3%)	1 (4.2%)	9 (37.5%)
Suffolk					
Total	27/75	16 (59.3%)	7 (25.9%)	2 (7.4%)	2 (7.4%)
Adult	26/59	15 (57.7%)	7 (26.9%)	2 (7.7%)	2 (7.7%)
Juvenile	1/16	1 (100%)	0 (0%)	0 (0%)	0 (0%)
Male	9/23	5 (55.6%)	2 (22.2%)	1 (11.1%)	1 (11.1%)
Female	18/52	11 (61.1%)	5 (28.8%)	1 (5.6%)	1 (5.6%)

**Table 4:** Human population density (residents per km^2^) and population levels within a 10 km radius of sampling locations.

Location	Population Within 10 km Radius	Residents per km^2^	No. Location/No. Total	No. Positive/No. Tested	Seroprevalence in % (95% CI)	% MAT 1:200
Onondaga						
Camillus	60,498	374	14/69	9/14	64.29 (35.14–87.24)	29%
Dewitt	185,776	389	19/69	12/19	63.16 (38.36–83.71)	21%
Fayetteville Solvay Syracuse	68,205 240,184 272,663	959 1355 3237	14/69 5/69 17/69	8/14 2/5 13/17	57.14 (28.86–82.34) 40.00 (05.27–85.34) 76.47 (50.1–93.19)	29% 0% 41%
Suffolk						
Islip	214,674	765	75/75	27/75	36.00 (25.23–47.91)	7%

**Table 5:** Limiting the impact of *Toxoplasma gondii*-infected white-tailed deer.

Develop and enhance public education and outreach programs to prevent *T. gondii* exposure when handling and consuming raw and undercooked venison.
Cook deer meat to a minimum of 71 °C (160 °F) or freeze for a minimum of 2 days at −12 °C to prevent foodborne infections.
Thoroughly wash and sanitize all surfaces, cutting boards, utensils and dishes that come in contact with raw meat to prevent cross-contamination.
Proper hand hygiene or wearing of gloves should be carried out whenever handling carcasses or raw meat.
House cats indoors to reduce environmental contamination.
Consider additional signage in areas of high *T. gondii* seroprevalence in WTD to reduce vehicular collisions.
Prioritize open county-wide hunts over controlled park hunts to reduce the impact of *T. gondii*-infected WTD.
Continue surveillance efforts across a rural to urban gradient to assess the local seroprevalence in *T. gondii*.

## Data Availability

The original contributions presented in this study are included in the article. Further inquiries can be directed to the corresponding author.
